# Early term ocular changes after cold compress application

**DOI:** 10.5935/0004-2749.20220031

**Published:** 2022

**Authors:** Alperen Bahar, Gökhan Pekel

**Affiliations:** 1 Ophthalmology Department, Yozgat State Hospital, Yozgat, Turkey; 2 Ophthalmology Department, Pamukkale University, Denizli, Turkey

**Keywords:** Glaucoma, Optic nerve injury, Eye injuries, Vasoconstriction, Cryotherapy/instrumentation, Tomography, optical coherence, Glaucoma, Traumatismo do nervo óptico, Traumastismo ocular, Vasoconstrição, Crioterapia/instrumentação, Tomografia de coerência óptica

## Abstract

**Purpose:**

To examine changes in the eyes after cold compress application at the early stage.

**Method:**

A total of 62 eyes from 62 healthy adult participants were included in this cross-sectional and comparative study. The subfoveal choroidal thickness and retinal nerve fiber thickness were measured by spectral-domain ocular coherence tomography. The central corneal thickness, anterior segment volume and depth, iridocorneal angle, and pupil diameter were measured by means of the Scheimpflug anterior segment imaging method. The measurements were repeated after 10 min of cold compress application, which was applied using special packs. The procedures were then repeated with non-cold packages to exclude the effect of pressure.

**Results:**

The average age of the participants was 30.74 ± 5.82 years. There was no significant change in the central corneal thickness after cold compress application, and there was a significant decrease in the anterior segment volume (p<0.001), anterior segment depth (p<0.001), and pupil diameter. Moreover, the iridocorneal angle increased (p=0.002). The subfoveal choroidal thickness decreased after the application of cold compress (p<0.001). The overall disk thickness (p=0.034) and superior nasal scale (p=0.007) significantly decreased after the cold compress was administered during the evaluation of optic nerve fiber thickness. In contrast to that with the cold application, the subfoveal choroidal thickness and optic nerve fiber thickness did not change after the non-cold compress application (p>0.05).

**Conclusion:**

Cold compress application may thus cause some physiological changes in the eyes, which necessitates the examination of its usage and effects.

## Abstract (pt)

**Objetivos:**

Examinar as mudanças nos olhos após a aplicação com compressa fria.

**Método:**

Sessenta e dois olhos de 62 adultos saudáveis foram incluídos neste estudo transversal e comparativo. A espessura da coróide subfoveal e a espessura da fibra nervosa retiniana foram mensuradas por tomografia de coerência óptica de domínio espectral (OCT). A espessura central da córnea, o volume e a profundidade do segmento anterior, o ângulo iridocorneano e o diâmetro da pupila foram mensurados por meio do método de imagem do segmento anterior de Scheimpflug. As medições foram repetidas após 10 minutos de aplicação de compressas frias, aplicadas com embalagens especiais. Os procedimentos foram repetidos com embalagens não frias para excluir o efeito da pressão.

**Resultados:**

A média de idade dos participantes foi de 30,74 ± 5,82 anos. Embora não tenha havido alteração significativa na espessura central da córnea após a aplicação da compressa fria, houve diminuição significativa no volume do segmento anterior (p<0,001), na profundidade do segmento anterior (p<0,001) e no diâmetro da pupila. Além disso, o ângulo iridocorneano aumentou (p=0,002). A espessura da coróide subfoveal diminuiu após a aplicação da compressa fria (p<0,001). A espessura total do disco (p=0,034) e a escala nasal superior (p=0,007) diminuíram significativamente após a administração da compressa fria durante a avaliação da espessura da fibra do nervo óptico. Ao contrário da aplicação com compressa fria, a espessura da coróide subfoveal e a espessura da fibra do nervo óptico não mudaram após a aplicação da compressa não fria (p>0,05).

**Conclusão:**

A aplicação de compressa fria pode causar algumas alterações fisiológicas nos olhos e o seu uso e efeitos devem ser observados.

## INTRODUCTION

Cold application is a proven treatment approach for soft tissue traumas^(1-4)^ and post-nerve injuries^(5,6)^ in terms of reducing inflammation and maintaining tissue viability. Inflammatory situations cause dilation at the vessels via the mediator. As a result, the interstitial fluid leakage from the blood vessels occurs. The vasoconstriction in the vessels after cold application leads to a reduction in leakage and consequently in the reduction of edema^(7,8)^. This finding supports the prevention of damage by slowing the metabolism through the ions and membrane potential in the neurons^(9,10)^.

Cold application is not a widely used method as a primary treatment in ophthalmology, but it is used for relief by patients in the face of diseases such as ocular allergic conditions and adenoviral conjunctivitis. Cold compress application packs have recently been produced with this purpose in mind. Some studies have suggested that it can be used to reduce the nerve destruction associated with optic neuropathy^(11)^ to reduce the complications of iridoplasty^(12)^ and in conjunction with analgesia before intravitreal injection^(13)^. In the present study, we aimed to observe the ocular changes after cold compress application. The main purpose of this research was to analyze the response of the eye to coldness, which has a choroid-rich vein network. It is possible that, subsequently, cold compress application may be used as a treatment method under the inflammatory conditions, such as retinal and uveal inflammatory conditions. In light of the data obtained, we can question the availability of cold compress application as a treatment method in case of acute angle closure.

## METHODS

The present study was initiated at our university hospital when we sought the permission of 64 healthy adult participants. A signed informed consent form was obtained from all participants after receiving an explanation about the nature and the possible consequences of the research at the time of study enrollment. Only the right eyes of the participants were studied. Two participants were excluded from our research due to the detection of eye disease (e.g., suspected glaucoma and central serous chorioretinopathy) at the initial stages. The study was conducted in line with the Declaration of Helsinki and approved by non-interventional clinical research ethics committee of the Pamukkale University.

### Study population

The participants were selected from healthy individuals of age 20-40 years. Before starting the study with all individuals, a standard eye examination was applied, which involved the use of an autorefractometer, a test of visual acuity, biomicroscopy, tonometry, and a retinal examination. Patients with a refraction defect of over ± 2.00 diopter, those having ocular surgery, and those with any ocular disease or systemic chronic disease were excluded. In all the participants, the corrected visual acuity was measured as 20/20 by the logMAR chart.

### Measurements

In the afternoon, the participants were examined while they were not hungry and had not consumed any caffeine. Optic disc nerve fiber imaging was performed in the presence of spectral-domain optical coherence tomography (OCT) (Spectralis^®^ software version 6.0, Heidelberg, Germany) as was choroid imaging in the simultaneously enhanced depth optical coherence tomography mode. Spectralis^®^, which takes 40,000 scans per second and utilizes the spectral-domain technique with a wavelength of 870 nanometer, was used for this purpose.

The choroidal thickness was measured manually using the instrument program (Heidelberg Eye Explorer 1.7.0.0) as the distance between the outer edge of the hyper-reflective retinal pigment epithelium from the subfoveal region and the inner edge of the sclera. Specific attention was paid to ensure that the image quality was sufficiently sharp for the scleral and choroidal tissues to distinguish them from each other. Repeats were performed when the image quality was unsatisfactory.

**Figure 1:**
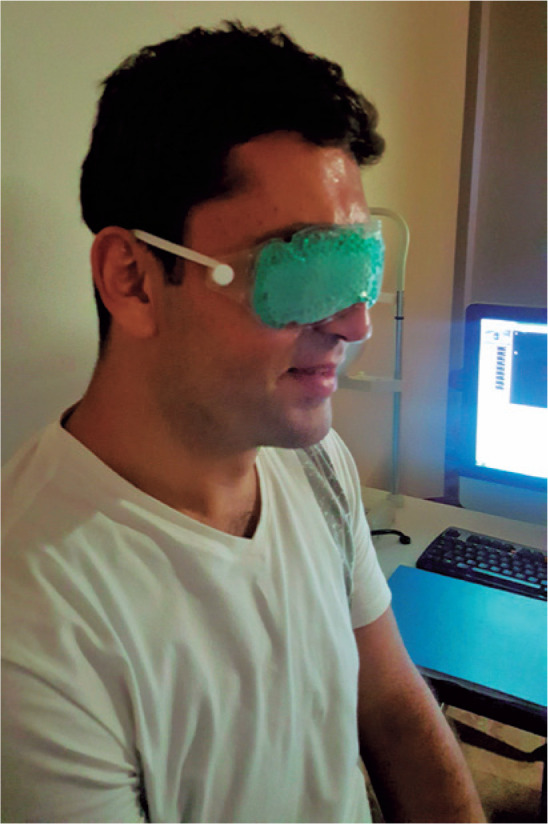
Participants subjected to the cold mask application (TheraPearl Eye-ssential Mask, Bausch & Lomb) for 10 min.

The measurements of anterior segment depth, anterior segment volume, iridocorneal angle, central corneal thickness, and pupil diameter measurements were performed with the Pentecam HR (Oculus, Wetzlar, Germany). Pentacam HR is a rotating Scheimpflug camera system that analyzes up to 50 high-resolution slit images for the frontal segment analysis. Scheimpflug images of the participants were examined by scanning with this system, and the repeats were performed for those whose image quality was insufficient. After all these measurements were made, the participants had the cold packs administered to them (TheraPearl Eye-ssential Mask, Bausch & Lomb) for 10 min (Figure 1) (The pack dimensions were 9 x 2.75 inches, and the pack was filled with pliable gel pearls). Before application, the mask was left in a freezer set to 0°C (32 Fahrenheit) for 2 h; the temperature was selected based on the recommendations of a similar study published by Lee et al.^(11)^. After the application, the previous measurements were repeated in the same order and under the same conditions. Meanwhile, during the application, pressure was applied to the eye, a factor that may have affected the said measurements. Consequently, the experiment was repeated after 1 week with the non-cold pack so as to distinguish between the effects of temperature and pressure. The measurements were made prior to the application, after which the non-cold pack was kept at the room temperature for 2 h before being applied for 10 min prior to the measurements being repeated. The measurements before the cold and non-cold compress applications were subsequently evaluated and did not reveal any significant difference between them. Therefore, the mean of the measurements before the applications was calculated and used in the statistical analysis as “*The Measurements Before the Applications”*.

### Statistical analysis

The statistical analysis was performed using the Statistical Package for the Social Sciences for Windows (Version 21.0, SPSS Inc., Chicago, IL, USA) after the data was transferred to the computer. The normality of data was examined using the Kolmogorov-Smirnov test, while the sample size was selected by taking a=0.05, b=0.20, and the standard effect size = 0.70 from the t-test table.

Analyses were performed using a paired sample *t*-test for before and after comparisons. P<0.05 were considered to be significant. Descriptive findings were expressed as mean ± standard deviation (SD).

## RESULTS

A total of 62 right eyes of 62 participants were included in the study. The average age (± SD) of the participants was 30.74 ± 5.82 years. A total of 32 of these participants were women (51.6%). The highest, lowest, and the average measurement values before the applications are shown in table 1.

**Table 1:** Highest, lowest, and average measurement values before cold pack application

Measurements (n=62)	Minimum	Maximum	Mean	Std. deviation
Central corneal thickness (µm)	491.00	605.00	540.66	29.80
Anterior segment volume (µL)	78.00	271.00	169.91	42.26
Anterior segment depth (µm)	1.98	3.71	2.94	0.40
Iridocorneal angle	25.60	50.40	36.95	6.47
Pupil diameter (µm)	2.29	5.04	3.24	0.57
Subfoveal choroidal thickness (µm)	169	519	333.72	83.93
Optical disk thickness (µm) (General)	72	141	104.98	11.75

A significant decrease in the anterior segment volume (p<0.001), anterior segment depth (p<0.001), and pupil diameter (p<0.001) were noted, although there was no significant change in the central corneal thickness after the cold compress application. The iridocorneal angle increased (p=0.002), while the subfoveal choroidal thickness decreased after cold compress application (p>0.001). After the cold pack was employed, the overall disc thickness (p=0.034) and superior nasal scale (p=0.007) were significantly reduced during the evaluation of optic disc nerve fiber thickness. The differences in other quadrants were however not significant.

Conversely, optic disc nerve fiber thickness (p=0.95) and subfoveal choroidal thickness (p=0.92) did not change after the non-cold compress application. For anterior segment measurements, similar to that in the aftermath of cold compress application, a significant decrease in the anterior segment volume (p<0.001), anterior segment depth (p<0.001), and pupil diameter (p=0.012) were noted. The iridocorneal angle was however increased (p=0.005).

## DISCUSSION

Applying a cold substance to an injured area is quite an old recovery and symptom relieving method practiced in the general medical. Nevertheless, there is no clear approach related to its usage in the field of ophthalmology. In this study, our goal was to observe how the use of cold packs impact the eyes. For this purpose, we investigated whether cold compress applications can reduce choroid and optic disc nerve fiber thickness and whether the pressure applied with the cold pack can lead to changes in the anterior chamber.

This research demonstrated the existence of a decrease in the choroidal thickness of the participants after cold compress application (p<0.001). However, no other changes were witnessed after the non-cold compress application. Thus, we believe that the said change was not a consequence of pressure, rather it was an effect of the colder temperature. Vasoconstriction of the blood vessels may be responsible for the decrease in the tissue thickness, including choroids where the blood vessels are extremely dense. Lee et al. noted that the temperature dropped to <37°C in the eye after 10 min of local cold compress application^(11)^. Furthermore, Shepherd et al. showed that cooling between 28°C and 37°C causes the vessels to contract by increasing the adrenoreceptor sensitivity^(7)^. As a result, the intraocular decline in the temperature after cold compress application may cause the vessels to contract. Moreover, Schaser et al. showed that capillary permeability is seriously reduced after cold application, which may contribute to the regulation of microcirculation^(2)^. As a part of their own study findings, Hodges et al. argued that there is a decrease in nitric oxide synthase activity after cold application and suggested it to be a contributing factor to vasoconstriction^(14)^. Khoshnevis et al. also reported that the vasoconstriction effect after cold application may continue even after the end of the application^(15)^. According to several different studies, central serous chorioretinopathy is abnormal in terms of microvasculature and circulation^(16,17)^. In fact, there is also a debate about whether the increase in nitric oxide concentration is an effect caused by the etiology of SSROP^(18)^. When scrutinizing all this data, it is reasonable to assume that cold compress application can contribute to treatment in pachycorrhoid cases, such as central serous chorioretinopathy (SSRP), where capillary permeability and choroidal thickening can occur.

From the results obtained in this study, we noted that cold compress application could reduce the thickness of the optic nerve fiber, while non-cold compress application had no such effect. Consequently, we believe that the decrease in the thickness of the optic nerve is dependent on temperature and that this decrease may be the result of changes in the vessels. Based on these fin dings, we demonstrated that cold and non-cold compress appli cation can similarly affect the anterior segment parameters. These effects did not probably result from the cold application, rather from the local pressure of the cold application pack, because there was no significant difference in the anterior segment parameters between these variables. It is indeed possible that the decrease in the anterior segment volume and the anterior segment depth may be due to the effect of pressure. An increase in the iridocorneal angle may also have occurred due to the pressure-resultant effect, which is similar to that of indentation. Meanwhile, Forbes demonstrated that the angle expanded and that the iris root was pushed backward after indentation^(19,20)^. There is therefore a need for additional research to determine whether the decrease in the pupil diameter is a resulting factor of the applied pressure or the effect of the administration of coldness. Considering the outcome of cold compress application on the anterior segment, it may be logical to use this treatment approach for acute angle closure glaucoma. In addition to the changes in the iris, the feeding of the optic nerve, which is adversely impacted by the increased effect of pressure during the glaucoma crisis and the protective effect of cold at ischemia, also possibly benefits.

On the flip side, besides the possible benefits, cold application can be harmful in some cases like flammer syndrome and open-angle glaucoma. Flammer syndrome refers to a phenotype characterized by the presence of primary vascular dysregulation together with a cluster of additional symptoms and signs^(21)^. Terelak-Borys et al. demonstrated that cold provocation induced a transient visual field deterioration in the glaucoma patients with flammer syndrome^(22)^. Vasospasm and elevated plasma endothelin-1 level have also been associated with glaucoma (especially normal tension glaucoma)^(23)^. In addition, Nicolela et al. showed that patients with glaucoma have an abnormal increase in their plasma endothelin-1 levels after the body cools down^(24)^.

The fact that the number of participants used in this study was less may be detrimental to the validity of our findings. The participants selected for our analysis were healthy people without any systemic/eye diseases. In the face of different illnesses, the results could have varied. The manual preparation of choroidal thickness analysis and the difficulties presented in ensuring standardization may also have affected the measurement outcomes. On the other hand, study-related hypotheses were set up according to the immediate effect of cold compress application, albeit this research did not examine the long-term effects of cold compress application.

In light of our study results, we noted that the cold application packs can induce physiological changes at the early stage in the eye structure including in the retina and choroid. Cold application is a frequently used treatment modality in the general medical literature, and there remains insufficient data available on its uses in ophthalmology. More analysis is therefore warranted to uncover how the resulting changes in the eye emerges and how these effects can be utilized in ophthalmology. Certainly, research relating to cold compress application on patients with different ophthalmologic diseases is required to further validate our findings.

## Figures and Tables

**Table 2:** Comparison of measurement values (values with a significant difference are shown in italics)

	The measurements before applications (n=62) (Mean ± SD)	After cold application (n=62) (Mean ± SD)	After non-cold application (n=62) (Mean ± SD)	P^a^ value
Central corneal thickness (µm)	540.66 ± 29.80	541.71 ± 29.80	540.95 ± 29.90	0.144
Anterior segment volume (pL)	**169.91 ± 42.26**	165.18 ± 40.47	165.80 ± 41.52	**0.000**
Anterior segment depth (mm)	**2.94 ± 0.40**	2.91 ± 0.39	2.90 ± 0.49	**0.000**
Iridocorneal angle	**36.95 ± 6.47**	38.43 ± 6.38	37.15 ± 6.42	**0.002**
Pupil diameter (mm)	**3.24 ± 0.57**	**2.93 + 0.47**	**3.02 ± 0.43**	**0.000**
Subfoveal choroidal thickness (pm)	333.72 ± 83.93	**313.09 ± 79.68**	330.92 ± 80.05	**0.000**
Optical disk thickness (pm)				
General	104,98 ±11.75	**104.50 ± 11.54**	104,92 ±11.75	**0.034**
Nasal superior	115.38 ± 24.98	111.93 ± 22.78	114.98 ± 24.98	**0.007**
Nasal	78.83 ± 12.70	77.93 ± 12.85	78.99 ± 12.65	0.098
Nasal inferior	117.98 ± 23.21	117.56 ± 21.56	117.97 ± 20.16	0.495
Temporal inferior	151.81 ± 25.68	151.80 ± 24.95	151.83 ± 22.65	0.935
Temporal	76.93 ± 17.67	76.25 ±17.17	76.91 ± 17.80	0.392
Temporal superior	144.16 ± 22.30	143.95 ± 20.65	144.10 ± 21.20	0.717

a = paired sample T-test; SD= standard deviation.
